# Supplementary material for: Predictors of neonatal mortality in the Eastern Regional Hospital in Ghana: A retrospective cohort study
Source: PLOS Glob Public Health. 2024 Jun 6;4(6):e0003295. doi: 10.1371/journal.pgph.0003295 (PMC11156434; doi:10.1371/journal.pgph.0003295)
Supplement: Attachment — Submitted filename: Response to Reviewers.docx [file pgph.0003295.s002.docx]

We thank the reviewers for their insightful comments. Their feedback on our manuscript has strengthened our manuscript.

**Reviewer #1:**

1. The efforts of the team are well appreciated. It is indeed an important topic.
2. While studying the predictors of mortality, rather than categorising the patient as walk in or born at eastern region hospital, it would be more prudent to categorise as born in hospital setting, home setting with no trained personnel, home setting with trained personnel, with or without delivery kits. That would give more information on why did the other group not born in eastern regional hospital has a higher hazards ratio of dying. This will make the conclusion more meaningful to a clinician. If this information is available or can be extracted, it should be added.

>>> We acknowledge the importance of categorising the data as born in hospital setting, home setting with no trained personnel, home setting with trained personnel, with or without delivery kits. However, we were limited to the data in the electronic medical records and so we did not have data on home delivery to make such a categorisation. We have added this statement to account for this in our limitation paragraph “This study was limited to the data in the electronic medical records of the hospital and therefore data on other factors, including potential confounders (e.g., timing on onset of symptoms to admission, household wealth, home delivery, etc.) were not available”.

3. The authors have reported in discussion that the mortality was lower in neonatal jaundice, and higher in asphyxia than neonatal sepsis. This finding should be read with caution as the study was not designed with the objective to study the mortality rates in different disease conditions and may not be powered enough. It would be better to use the phrase “our study observed” rather than “our study found”. This should also not be a part of conclusion. At the most it can say that which group had the highest hazards of dying and which group had the lowest.

>>> We have implemented your suggestions both in discussion and conclusion sections.

4. The last conclusion, “We recommend the implementation of interventions that would ensure prompt and high-quality neonatal care to avert mortalities from the complications that occur in newborns” cannot be concluded or recommended from the current study as the study neither has defined any intervention, nor it has tested it.

>>> We agree with you, and this has been removed.

Minor comments
1. Line 137: Majority seems a wrong word to describe 65.1% of population

>>> This has been changed to “A greater proportion.”
2. Line 139: Years is missing after 27.1

>>> This has been added.
3. There are minor grammatical errors through the manuscript that need to be corrected.

>>> We have read the manuscript and addressed the grammatical errors.

**Reviewer #2:**

1. Authors may consider adding a time component in the title to give the audience a clear message regarding the study's timeline.

>>> We prefer not having a time component in our title.

2. The authors used secondary data from the EHR of the hospital belonging to one episode of admission and outcome among the neonates. Therefore, the study design claimed in the paper (retrospective cohort study) is debatable. Since there was no follow-up period and outcomes were during the same episode of admission, the authors may consider the design as a cross-sectional analytical study using the secondary data from EHR rather than a retrospective cohort.

>>> This is not a cross-sectional study as the outcome and exposures (i.e., characteristics) were not assessed at one point in time. Our study was retrospective cohort study. There was a follow-up period, which was retrospective (i.e., following back in time). No neonate had our outcome of interest (i.e., death) at the time they were admitted (i.e., time point 0). Each neonate spent some time (unit of measure was day) in the neonatal intensive care unit before our outcome of interest was observed (death versus censored). This is a time to event analysis based on the time each neonate was admitted until the time our outcome of interest was observed.

3. All the predictors identified in the study were already known factors and didn't add anything new to the existing literature. The authors can rework the discussion part to bring out any further information added to the scientific literature from this analysis.

>>> We agree that all predictors are known in literature. However, there are limited studies in Ghana on this subject, particularly in the Eastern Region. One of the reasons of this study was to guide and inform healthcare workers and policy makers in the Eastern Region to have a situational understanding of predictors of neonatal mortality in their region. We have added additional text on the rational/purpose of the study. We compared our findings to appropriate literature in the discussion.

4. The paper is predominantly in passive voice. The authors can consider writing in an active voice, which will bring more clarity to the sentences.

>>> We have read the manuscript and addressed it.

Abstract

5. The abstract fails to bring out the rationale of the study.

>>> Because we are mindful of exceeding the abstract word count, we did not include the rational. However, we are of the view that the aim of the study in the introductory part of the abstract is appropriate.

6. The abstract's methods part should clearly show that this is a secondary data analysis using the existing EHR data of Hospital X from Y to Z time stating the critical exposure and study outcomes.

>>>We have addressed part of your comment and indicated that the data was secondary data from electronic medical records. The exposures and outcome can be inferred from the introduction and the result sections of the abstract. Addition of exposures and outcome will exceed the abstract word count.

7. The authors try to show all the results in the abstract. Instead, they can limit the key findings alone and try to focus more on the methods part.

>>> This was an exploratory analysis of predictors of neonatal mortality using maternal and neonatal characteristics. All results in the abstract were significant and key to the aim of the study. We also think that the method section of our abstract has the main features (i.e., study design type, type of data, source and analysis conducted).

8. The recommendations written in the conclusion are very broad. The study doesn't show any results in the implementation status of neonatal care in the hospital but only finds the predictors of mortality. Authors can rethink making the recommendations more specific on the lines of "process evaluation of the neonatal care delivery" and the antenatal care program delivery to ensure 100% compliance. Also, the authors can recommend looking at "why the antenatal care program is not able to provide care for x% of the mothers?"

>>> We agree with you that our “study doesn't show any results in the implementation status of neonatal care in the hospital but only finds the predictors of mortality.” We have reworded our conclusion to read “Averting complications such as low birthweight, hypothermia, hyperthermia, birth asphyxia, including the provision of obstetric and early neonatal care within the first 24 hours of life is critical to reducing neonatal mortality.”

We now recommend for further studies to evaluate neonatal care delivery as well as antenatal care program in the Eastern Region. This was added to the conclusion section of the manuscript.

Introduction

1. It appears that there is already an existing program for addressing neonatal mortality. The rationale is not very clear as the study here is trying to look at the factors, which are already established risk factors for neonatal mortality.

>>> There is a Newborn Health Strategy to improve neonatal care in the Country. We have added additional text to the rational of the study in the last paragraph which now read as follows: “Research on factors associated with neonatal mortality in Ghana has largely been conducted among the general population, while fewer studies have been conducted in hospital settings. Many of these studies did not employ a robust design. Literature on predictors of neonatal mortality in the Eastern Regional Hospital is also lacking. The goal of this study was to assess the predictors of neonatal mortality at the Eastern Regional Hospital in Ghana using a retrospective cohort design. Findings from this study will inform decision and policy making with regards to reducing neonatal mortality in Ghana, particularly in the Eastern Region.”

2. The time component is missing in the study's aim.

>>> We have the time component in the methods sections.

Methods

3. The authors claim this study to be a retrospective cohort which means that the birth details were extracted from the past timeline and followed them up to quantify their outcomes. However this information is missing in the methods section. What was the frequency of follow-up? Who followed them up? The author team may consider this a cross-sectional analytical study using EHR-based secondary data.

>>> See our earlier response explaining why this is clearly a retrospective cohort study. This is not a cross sectional study as the exposures and outcome were not measured at one point in time. Additionally, the neonates were admitted and being treated by health workers overtime before our outcome of interest was observed. We analysed secondary data so we couldn’t have followed them up. We don’t expect a frequency of follow-up because we analysed secondary data unlike a prospective cohort study where the researchers are in control.

4. Details on how the data were entered into the Electronic Health Record (EHR) need to be provided.

>>> We extracted the data from electronic medical records of the hospital. The electronic medical records/ electronic health records of the hospital are generated when health workers input data of patients, including neonates into computerized system designed for entering patient data in the hospital. We think that it is not necessary to include this information on how data are entered into the Electronic Health Record (EHR) of the hospital.

4. The authors may add details regarding the admission pattern, accessibility of the hospital, number of beds in the NICU, facilities available, including workforce and equipment, etc, to the methods section to provide the context for answering the research question. Is this the highest facility available in the region (this can affect the admission pattern)? Description of the setting is important.

>>> We have added text to address the admission pattern, accessibility of the hospital, number of beds in the NICU, workforce and equipment.

5. The authors claim that the study's primary outcome was time to death. But the primary outcome is the status of the neonate at a specified period – dead or alive. The team may revisit the paragraph on the outcome to bring this out clearly.

>>> This is a time to event analysis (i.e., survival analysis). Our outcome is not dead or alive. A survival analysis takes into account time and censoring as described in our outcome subsection in the methods section as “The primary outcome was time to death from when the neonate was admitted to the NICU of the Eastern Regional Hospital. The outcome was dichotomized as “1” if the neonate had developed the event (i.e., died) and “0” if the neonate was censored. Censoring in this study occurred if the neonate recovered and was discharged or if the neonate did not experience the event before the study period ended.”

6. What software did the study team use for analysis? Kindly provide the same with adequate reference.

>>> We have added this.

7. What is the source of the live births data for calculation of NMR? This should be clearly stated in the methods section.

>>>The NMR was estimated from our secondary data extracted from the electronic medical records This was characterised as the probability of dying during the first 28 days of life expressed per 1,000 live births. We have added this to the methods section, specifically the data analysis section.

8. The methods section should specify the calculation of unadjusted HR, and then adjusting using the Cox regression model should be clearly stated. In the current version, the author explains these in the results section. The author may mention all methods, tests used, etc, in the methods section.

>>> We clearly stated all tests or analytic methods used in the methods section. The univariate and Cox multivariable regression represents unadjusted and adjusted HRs respectively. How the univariate and Cox multivariable regression analyses were conducted can be found in our data analysis section.

Results

9. The results section starts with the line – "a sample of …". Did the study team perform any sampling for this study? From my understanding of the results – they included all the admissions to NICU during the study period (no sample). The team can consider rewriting this line.

>>> We have reworded the sentence to read “Data for 1684 neonates were analyzed.”

10. The percentages written in the parenthesis are confusing. In line 142, the authors say – Among the neonates who died, 73 were premature. (73/199 should be around 36%, but it's written 4.4%). Authors can clearly state the denominators used. Similar issue in line 143 also (167/199??).

>>> We have reworded it to read “The number of diagnosed of prematurity on admission were 73 (4.4%)” to avoid the confusion. The denominator is 1654, which is 1684 -30 (i.e., missingness for this variable). The sentence following “Most of the deaths, 167 (9.9%) occurred within the first 24 hours after birth” makes sense now, which has a denominator of 1684.

11. The author may save words by avoiding mentioning the tests (line 144) used for comparisons in the results section. It's already mentioned in the methods section.

>>>We understand your comment, but we introduced it to describe what the figures represent.

12. In Table 1, the parenthesis value issue exists. Generally, in a cohort study, we try to show row percentages. But here it's all overall percentages. The authors may reconsider this.

>>> We think that the presentation of our Table 1 is appropriate. The first column is the characteristics (i.e., exposures), which show the percentages for categorical variables. More importantly for our survival analysis, we show the absolute numbers and proportions for neonates that survived versus died for varies categories of our characteristics.

13. Kindly consider reducing the p-value to three decimal places and the ink-to-data ratio in all tables.

>>> We have reduced the P-values to three decimal places in the Tables.

14. Figures 1 and 2 are not clear in the reviewer copy. Kindly submit high-quality images for publication consideration.

>>> We acknowledge your concern. We guess it’s the copy you got as a reviewer. Our figures are according to the dimensions and resolution recommended by PLOS Global Public Health and are high quality for publication.

15. Data Quality is an essential component in secondary data analysis. The authors may comment on this in the results section. Was there only 1684 admissions? Were there any duplicates – if yes, how did you handle the same should be explained.

>>> We acknowledge the importance of your concern, but we did not have duplicates. We only had missing data for diagnosis on admission and multiple gestation, which did not warrant imputation because of the low percentage of missingness.

Discussion

16. The discussion section can improve a lot. The first paragraph can be the key findings alone –a maximum of three take-home messages. An in-depth discussion of these critical findings follows them. However, in the current version – the first paragraph is too detailed with comparisons to other studies. The author team may consider cleaning it up to reveal two or three key messages.

>>> We have rewritten the first paragraph, which provides a summary of the key results.

17. Also, after making necessary comparisons with existing studies, the author team should be able to interpret the findings for the current settings (conclusion and recommendation for that finding). The current version of the manuscript is deficient in this.

>>> We have compared our findings to existing studies, including given possible reasons why we observed each of our key findings. We did not include conclusions for each paragraph as we limited that to the conclusion section of the manuscript.

18. In lines 227-230, the team can speak about the type and level of facilities –a significant factor that can affect mortality (the author team should add the details of the current health facility in the methods and discuss in detail in the discussion).

>>> We have added text to this paragraph in the discussion to explain why the mortality was lower in our study compared to many other referral facilities in Ghana. We also did address the admission pattern, accessibility of the hospital, number of beds in the NICU, workforce and equipment in the methods section, which provides further insight to our observed finding.

19. In lines 237-238, the author's team discusses using these results for policy and interventions. The team can make it more specific than being broad. For example, the maximum deaths are due to prematurity. In future studies, we can try to understand why premature births occur and then act on those – which could be a more specific recommendation. Also, the team can suggest a program evaluation to understand the quality of care being delivered at the facility.

>>> We have reworded it to make the policy and interventions targeted at strengthening neonatal care and antenatal care programs. We have also recommended for further studies to consider evaluating the process of neonatal care delivery as well as antenatal care program in the Eastern Region to assess why most mothers did not meet the WHO recommendation. This was added to the conclusion section.

20. The recommendations written in the conclusion part are very broad. The study doesn't show any results in the implementation status of neonatal care in the hospital but only finds the predictors of mortality. Authors can rethink making the recommendations more specific on the lines of "process evaluation of the neonatal care delivery" and the antenatal care program delivery to ensure 100% compliance. Also, the authors can recommend looking at "why the antenatal care program is not able to provide care for x% of the mothers?"

>>> We agree with you and have removed the recommendation. We now recommend for further studies to consider evaluating the process of neonatal care delivery as well as the antenatal care program in the Eastern Region to assess why most of them did not meet the WHO recommendation of eight or more antenatal care contacts.

21. The authors can conclude with what they think as a further study on using this data for decisions. The study team can also propose a qualitative approach to understanding why women don't get antenatal treatment.

>>> We have addressed this.
