# Supplementary material for: Predictors of neonatal mortality in the Eastern Regional Hospital in Ghana: A retrospective cohort study
Source: PLOS Glob Public Health. 2024 Jun 6;4(6):e0003295. doi: 10.1371/journal.pgph.0003295 (PMC11156434; doi:10.1371/journal.pgph.0003295)
Supplement: Attachment — Submitted filename: Response to Reviewers.docx [file pgph.0003295.s003.docx]

**Thank you for your comments. This has improved our manuscript.**

1. Line no 65, 66 - the word steady means decline is good. I think the authors mean that the decline is slow rather than steady.

>>> We have changed the word from “steady” to “slow.”

2. This study is conducted on the very high risk category on neonates admitted to the NICU. Justification for the inclusion criteria needs to be included in the introduction.

>>> We have addressed it in the last paragraph of the introduction section. It reads as follows “This study focused on neonates admitted in the neonatal intensive care unit (NICU) of the Eastern Regional Hospital as findings from this study will inform decision and policy making with regards to reducing neonatal mortality in the NICUs in Ghana, particularly in the Eastern Region.”

3. Was sample size calculation attempted? It would be good to state the calculated sample size to validate that the study was powered to identify the associations identified.

>>> We have now added a paragraph to state the minimum sample size, which was required for our study. It reads “To estimate the minimum sample size required for this study, a previously reported prevalence of neonatal mortality (20.2%) from a teaching hospital in Ghana was used [16]. Using a 5% margin of error and a 95% confidence interval, an estimated minimum sample size of 248 was required.”

4. Discussion can focus more on the higher mortality among the referred neonates, adding the existing level of care at the primary level of public health system and the importance of strengthening it in the recommendations.

>>> We have added text to expand our discussion on mortality regarding neonates who were referred. We have also added a recommendation to the conclusion section to strengthen primary healthcare so as to reduce mortality associated with neonates who are referred.
